# Temporal trends in hepatitis B and C infection in family blood donors from interior Sindh, Pakistan

**DOI:** 10.1186/1471-2334-8-43

**Published:** 2008-04-10

**Authors:** Syed Abdul Mujeeb, Mark S Pearce

**Affiliations:** 1 Associate Professor/Blood Bank Officer, Jinnah Postgraduate Medical Centre, Karachi, Pakistan; 2 Lecturer in lifecourse epidemiology, Institute of Health and Society/School of Clinical Medical Sciences, Newcastle University, Newcastle upon Tyne, UK

## Abstract

**Background:**

Hepatitis B (HBV) and C (HCV) infections are a serious global and national public health problem. Earlier studies have reported increasing rates of hepatitis infection in Pakistan, particularly in rural areas. Pakistan has no active surveillance program to monitor trends of these infections. The objective of this study was to verify this trend in blood donors from the rural Sindh area of the country.

**Methods:**

The study analysed the data of blood donors of interior Sindh who donated blood at JPMC blood bank from January 1, 2004 to September 15, 2007. HBsAg status was determined by using HBsAg Serodia kit and antibodies to HCV using the Detect HCV ™ V.3 Kit. Samples repeatedly reactive for HBsAg or anti-HCV were considered positive for HBV or HCV infection respectively.

**Results:**

The overall seroprevalence of HBV infection among donors was 6.2 % (95% CI 5.5%–6.9%) and did not change significantly over the study period. Overall seroprevalence of HBV infection in literate blood donors was 5.7 %(95% CI 4.7%–6.8%). Prevalence decreased significantly in this group over the study period (p = 0.05). No other significant trends in seroprevalence of HBV infection were seen in the stratified analyses.

The overall seroprevalence of HCV among donors was 7.5% (95% CI 6.8%–8.3%) and increased significantly over the study period from 7.2% (95% CI 5.8%–8.7%) in 2004 to 8.9% (95% CI 7.4%–10.6%) in 2007 (p = 0.02). Significant increase in seroprevalence was particularly seen in literate (p = 0.03), non–first time (p = 0.01) and Sindhi speaking (p = 0.01) donors.

**Conclusion:**

Our study finds a steady increase in the prevalence of HCV infection in blood donors from interior Sindh between 2004 and 2007. On the contrary, decreasing prevalence of HBV was found, particularly in literate blood donors. There may be a need to have rural community-based epidemiological studies to identify the determinants of the spread of HCV infection and also those that are limiting the spread of HBV infection particularly in the literate blood donor population.

## Background

Hepatitis B (HBV) and C (HCV) infections are a serious global public health problem. Worldwide, over two billion people have been infected with HBV and more than 350 million have chronic HBV infection [1]. An estimated 170 million people are chronically infected with HCV and 3–4 million people are newly infected each year [1,2].

HBV and HCV infections are also a serious public health concern in Pakistan. In a community-based study in Hafizabad, Punjab, HBV infection was prevalent in 4.3% and HCV infection in 6.5% of the residents [3]. Previous studies in Pakistan have reported that 20% of paid blood donors [4], 2.4% of replacement blood donors [5], and 1% of voluntary blood donors [6] had HCV infection, while 10% of paid donors and 5% of replacement donors had HBV infection [7]. In the northern part of the country, 2.5% of blood donors have HBV and 5.1% HCV infection [8]. Recently Alam reported increasing rates of HBV infection in Pakistan, and a strong association with residing in a rural area. He attributed lack of proper health facilities, deprived socio-economic status, and less public health awareness about the transmission of major communicable diseases as potential explanations for increasing rates of diseases such as HBV, HCV and HIV infection in the country. In particular, it was stressed that more studies were required to have a better understanding of the epidemiology of HBV infection in Pakistan [9].

Although in Pakistan both HCV and HBV are regarded as diseases of public health importance, no active surveillance program is available to verify the previous claims of increasing prevalence of hepatitis B and C infection, particularly in rural areas of the country. In the absence of such a program the Jinnah postgraduate medical centre (JPMC) blood bank, Karachi, as one of the largest blood banks operating in the country, provides a cost-effective method for monitoring the prevalence, pattern of distribution, and trends of these diseases. We previously reported the results of a baseline analysis of blood donors at the JPMC blood bank as part of a pilot phase to develop a sentinel surveillance system for HBV and HCV infections [10]. The present study addressed whether there has been an increase in the prevalence of HBV and HCV infections in a rural part of Pakistan. We selected family blood donors from the interior Sindh province to study the prevalence of HBV and HCV infection and monitor temporal trends between 2004 and 2007. Family blood donors are the family members of a patient in need of transfusion who donate blood and suffer relatively less selection bias compared with voluntary and paid blood donors.

Sindh, one of four provinces in Pakistan, has a population of 30,439,893 people. The proportion of the population living in rural areas is 51% [11]. However, excluding Karachi, the largest city in Pakistan with a population of over nine million, the remainder of Sindh province (interior Sindh) has a rural proportion of 74% [11].

## Methods

The study used data from the JPMC blood bank database. Only blood donors who donated blood at this facility from January 1, 2004 to September 15, 2007 and also reported their permanent and present address in interior Sindh (i.e. excluding Karachi) were included. The JPMC blood bank is a hospital based blood bank in the public sector. Its screening laboratory is affiliated with National reference laboratory, Australia (NRL) for quality assessment. A structured questionnaire was given to every literate donor to collect information on age, sex, literacy level, mother tongue, frequency of donations, and place of residence. Face to face interviews were used to collect information from illiterate donors using the same structured questionnaire. All reactive blood donors who collected their reports were provided counselling, further information and appropriate referrals. In routine practice, all paid donors or donors having high-risk behaviour or conditions were excluded from providing donations. The dataset included all the consenting healthy adults who passed routine pre-donation screening criteria and donated blood at the blood bank.

For screening of blood, a five-millilitre blood sample was collected aseptically into a sterile test tube from each donor and tested for HBsAg and anti HCV antibodies within 24 hours of collection. HBsAg status was determined by using HBsAg Serodia kit (Fujirebio. Inc.Tokyo, Japan). The screening assay has 99% sensitivity and 100% specificity [12]. Antibodies to HCV were determined using the Detect HCV ™ V.3 Kit (Adaltis Inc., Montreal, Canada). According to their package insert (2002), the screening assay has 98.2% sensitivity and 99.7% specificity.

Samples positive for any of the above were re-tested. Samples repeatedly reactive for HBsAg or anti-HCV was considered positive for HBV or HCV infection respectively.

This study was approved by the ethical committee of Jinnah postgraduate medical centre (JPMC), Karachi, Pakistan.

### Statistical analysis

Each entry of the dataset was verified from the original manual dataset. It was cleaned, validated, and analyzed using EP Info 6 (version 6.04; Center for disease control and prevention, Atlanta, GA, USA).

The study population was stratified by year of donation, age at time of donation (17–26 years or 27–65, using median age as a cut off point), sex, mother tongue (Sindhi, or other), level of literacy (illiterate or literate), and frequency of donation (first time or second time or more). Individuals with any missing data on the variables of interest were excluded from all analyses. Descriptive statistics were used to describe the data, and prevalence was calculated along with corresponding 95% confidence intervals (95% CI). Chi square test for trend was used to measure whether variations across groups were due to a trend.

## Results

During the study period, there were 5345 blood donations from donors eligible for this study and response to the questionnaire was excellent (>99%). Males overwhelmingly (99%) dominated the donor population (Table 1). The median age of the donors was 26 years (range 17–63). The average level of illiteracy in donors was 61%, ranging from 56% in 2005 to 67% in 2007. The overall proportion of Sindhi speaking donors was 76%, ranging from 70% in 2006 to 74% in 2004. The average proportion of first-time donors was 48%, ranging from 42% in 2005 to 54% in 2007.

**Table 1:** Characteristics of the study population by year of donation.

Variables	2004 N (%)	2005 N (%)	2006 N (%)	2007 N (%)	Total N (%)
**Sex**					
Male	1326(99)	1539(98.6)	1095(98.5)	1317(98.8)	5277(98.7)
Female	13 (1.0)	22 (1.4)	17 (1.5)	16(1.2)	68 (1.3)

**Age**					
17–26 Yrs	671(50.3)	794(51.1)	592(53.7)	696(52.4)	2753 (51.5)
27–65 Yrs	662(49.7)	760(48.9)	511(46.3)	632(47.6)	2565 (48.2)
Missing					27

**Literacy**					
Illiterate	823(61.9)	878(56.4)	663(60.2)	886(66.8)	3250 (60.8)
Literate	507(38.1)	678(43.6)	439(39.8)	441(33.2)	2065 (38.6)
Missing					30

**Mother tongue**					
Sindhi	995(74.4)	1250(80.1)	780 (70.1)	1048(78.6)	4073(76.2)
Others	343(25.6)	311(19.9)	333(29.9)	284(21.3)	1270(23.8)
Missing					2

**Type of donor**					
First time	668(50.0)	661(42.3)	535(48.1)	718(53.9)	2582(48.3)
Second or more	668 (50.0)	900(57.7)	577(51.9)	614(46.1)	2759 (51.6)
Missing					4

**Total**	**1339(25.1)**	**1561(29.2)**	**1112(20.8)**	**1333(24.9)**	**5345**

### Seroprevalence of HBV infection

The overall seroprevalence of HBV infection among donors was 6.2% (95% CI 5.5%–6.9%) and did not change significantly over the study period (Table 2). Similar results were seen when restricting the analysis to males. The number of female donors in this sample was too small to warrant statistical testing.

**Table 2:** Annual seroprevalence rates and linear trend analysis of HBV infection in blood donors of interior Sindh

**Variables**	**2004**	**2005**	**2006**	**2007**	**Overall Prevalence**	**Chi Square test for trend p**
**Annual prevalence**						0.33
%(n*)	7.5(100)	5.4(85)	5.4(60)	6.4(85)	6.2(330)	
95% CI	6.1–9.00	4.4–6.7	4.1–6.9	5.1–7.8	5.5–6.9	

**Sex**						
Male %(n)	7.5(100)	5.5(85)	5.4(59)	6.5(85)	6.2(329)	0.51
95% CI	6.2–9.1	4.4–6.8	4.1–6.9	5.2–7.9	5.6–6.9	
Female % (n)	0.0(0)	0.0(0)	5.9(1)	0.0(0)	1.5(1)	-

**Age**						
17–26 years %(n)	7.5(50)	5.7(45)	5.6(33)	5.7(40)	6.1(168)	0.25
95% CI	5.6–9.7	4.2–7.5	3.9–7.7	4.1–7.7	5.2–7.1	
27–65 years %(n)	7.6(50)	5.3(40)	5.3(27)	7.1(45)	6.3(162)	0.82
95% CI	5.7–9.8	3.8–7.1	3.5–7.6	5.2–9.4	5.4–7.3	

**Literacy**						
Illiterate % (n)	7.0 (58)	6.3 (55)	5.3 (35)	7.3(65)	6.6(213)	0.93
95% CI	5.4–9.0	4.8–8.1	3.7–7.3	5.7–9.3	5.7–7.5	
Literate % (n)	8.3 (42)	4.4 (30)	5.7 (25)	4.5(20)	5.7(117)	0.05
95% CI	6.0–11.0	3.0–6.3	3.7–8.3	2.8–6.9	4.7–6.8	

**Mother tongue**						
Sindhi % (n)	7.4(74)	6.1(76)	5.8(45)	6.0(63)	6.3(258)	0.23
95%CI	5.9–9.2	4.8–7.6	4.2–7.6	4.6–7.6	5.6–7.1	
Others % (n)	7.6(26)	2.9(9)	4.5(15)	7.7(22)	5.7(72)	0.88
95% CI	5.0–10.9	1.3–5.4	2.5–7.3	4.9–11.5	4.5–7.1	

**Type of donor**						
First time % (n)	8.4(56)	5.4(36)	6.2(33)	6.7(48)	6.7(173)	0.36
95% CI	6.4–10.7	3.8–7.4	4.3–8.6	5.0–8.8	5.8–7.7	
Second or more times % (n)	6.6(44)	5.4(49)	4.7(27)	6.0(37)	5.7(157)	
95% CI	4.8–8.7	4.1–7.1	3.1–6.7	4.3–8.2	4.9–5.6	0.58

* Total no. of HBV infected blood donors

Overall seroprevalence of HBV infection in literate blood donors was 5.7% (95% CI 4.7%–6.8%). Prevalence decreased significantly in this group over the study period (p = 0.05). No other significant trends in the seroprevalence of HBV infection were seen in the stratified analyses.

### Seroprevalence of HCV infection

The overall seroprevalence of HCV among donors was 7.5% (95% CI 6.8%–8.3%) and increased significantly over the study period from 7.2% (95% CI 5.8%–8.7%) in 2004 to 8.9% (95% CI 7.5%–10.6%) in 2007 (p = 0.02) (Table 3). Similar results were seen when restricting the analysis to males, with a slightly more significant linear trend (p = 0.01).

**Table 3:** Annual seroprevalence rates and linear trend analysis of HCV infection in blood donors of interior Sindh

	**2004**	**2005**	**2006**	**2007**	**Overall prevalence**	**Chi Square test for trend p**
**Annual Prevalence**						
%(n*)	7.2(96)	5.7(89)	8.8(98)	8.9(119)	7.5(402)	0.02
95% CI	5.8–8.7	4.6–7.0	7.2–10.6	7.5–10.6	6.8–8.3	

**Sex**						
Male % (n)	7.1(94)	5.7(87)	8.8(96)	8.9(117)	7.5(394)	0.01
95% CI	5.8–8.6	4.6–6.9	7.2–10.6	7.4–10.6	6.8–8.2	
Female % (n)	15.4(2)	9.1(2)	11.8(2)	12.5(2)	11.8(8)	0.94

**Age**						
17–26 years %(n)	5.2(35)	4.5(36)	8.1(48)	6.8(47)	6.0(166)	0.06
95% CI	3.7–7.2	3.2–6.2	6.0–10.6	5.0–8.9	5.2–7.0	
27–65 years %(n)	9.1(60)	7.0(53)	9.6(49)	11.4(72)	9.1(234)	0.08
95% CI	7.0–11.5	5.3–9.0	7.2–12.5	9.0–14.1	8.0–10.3	

**Literacy**						
Illiterate % (n)	8.6(71)	6.9(61)	10.1(67)	9.8(87)	8.8(286)	0.16
95% CI	6.8–10.8	5.4–8.8	7.9–12.7	7.9–12.0	7.8–9.8	
Literate % (n)	4.7(24)	4.0(27)	6.8(30)	7.3(32)	5.5(113)	
95% CI	3.1–7.0	2.6–5.7	4.7–9.6	5.0–10.1	4.5–6.5	0.03

**Mother tongue**						
Sindhi % (n)	7.1(71)	6.1(76)	9.2(72)	9.4(98)	7.8(317)	0.01
95% CI	5.6–8.9	4.8–7.6	7.3–11.5	7.7–11.3	7.0–8.6	
Others % (n)	7.3(25)	4.2(13)	7.8(26)	7.4(21)	6.7(85)	0.59
95% CI	4.8–10.6	2.2–7.0	5.2–11.2	4.6–11.1	5.2–8.2	

**Type of donor**						
First time % (n)	7.8(52)	7.7(51)	9.9(53)	8.5(61)	8.4(217)	0.44
95% CI	5.9–10.1	5.810.0	7.5–12.8	6.6–10.8	7.4–9.5	
Second or more times % (n)	6.6(44)	4.2(38)	7.8 (45)	9.3(57)	6.7(184)	
95% CI	4.8–8.7	3.0–5.7	5.7–10.3	7.1–11.9	5.8–7.7	0.01

* Total no. of infected blood donors

Increases in HCV seroprevalence were seen in both age groups, although not quite reaching statistical significance for either group. Significant increases in seroprevalence were also seen in literate (p = 0.03), non-first time donors (p = 0.01) and Sindhi speaking (p = 0.01) donors.

## Discussion

Our study finds a steady increase in prevalence in HCV infection in blood donors from interior Sindh between 2004 and 2007. This increasing trend of HCV infection is apparent in almost in all sub-populations of blood donors from interior Sindh. However, it becomes statistically more significant in male, literate, Sindhi speaking, and repeat blood donors. These findings are consistent with an earlier study conducted in Karachi during the period 1998–2002 which also found a linear increase in the prevalence of HCV infection in blood donors [13].

This study was conducted in a single institution, so results may have been influenced by characteristics of the donor population, specific practices in donor recruitment or sensitivity and specificity of the screening assays used. Therefore, results may not be generalisable to other segments of the society or the population.

A further limitation of this study was the inability to trace repeat blood donors and thus it is possible that some individuals may have been included more than once, resulting in either over or under estimation of the association, particularly in three or more time donors. However, this limitation may not be very significant as regular voluntary blood donors were excluded from the study and blood donors from interior Sindh mainly donate blood in the facility when they have a family member admitted in the hospital and require blood transfusion. Further, the trend in first time blood donors was not statistically different from that seen in repeat blood donors. No confirmatory tests were performed for HCV and HBV infections. It is possible that some false positive blood donors may be reported as positive in the study. However, despite this limitation, positive predictive values of the tests performed will remain high, due to the high prevalence of HCV and HBV infections in the study population.

Some previous studies have reported associations between HCV infection and unsafe injection practices in Pakistan [3,14]. It is quite likely that in rural areas the growing use of the allopathic health care system is contributing to the spread of HCV infection by increasing the use of unsafe injections and poor infection control practices. Increasing rates of HCV infection in literate blood donors is a disturbing finding and may reflect a more contextual effect of high prevalence of the infection in the area. Increasing rates of HCV infection in repeat donors is another interesting finding, particularly as any potential donor with a previous history of blood infection is requested to refrain from donating blood. It is quite likely that they might have donated blood earlier in some other facilities and remained ignorant of their HCV status and thus continued their practice of donation of blood. We did not find a similar increase in HBV infection in the blood donor population from Interior Sindh. On the contrary we found an apparent lowering trend of the infection, particularly in literate blood donors. This may be because being educated they are more health conscious. The recent introduction of HBV immunization is unlikely to be a major cause of reduction of HBV infection in the blood donor population because of the high median age of the study population.

A similar trend analysis study conducted in the Karachi blood donor population from 1998–2002 reported a statistically significant downward trend of HBV infection [15]. It is hoped that this lowering trend of hepatitis B infection will be more pronounced in the future in the younger age group in particular because of introduction of universal HBV vaccination for neonates through expanded program of immunization with the assistance of Global alliance for vaccines and immunization (GAVI) [16].

Following introduction of HBV immunization and increased availability and accessibility of HBV immunization for the general masses, we can expect some positive effect in controlling HBV infection. However, in situations where poor injection, blood transfusion and infection control practices are prevalent [17-19], a selective trend in the spread of HCV infection in both urban and rural settings is both interesting and alarming, particularly when risk of transmission of HCV infection from contaminated needles is 1.8% and the risk of HBV infection is 30%–60% [20].

A reason for the decrease in HBV prevalence amongst the donor population may be partially due to the fact that a healthy adult when exposed to HBV infection recovers completely in 95% of the cases, whereas they remain infected in 85% of the cases if exposed to HCV infection [21,22]. It may also be because the HBsAg marker used to screen for HBV infection is a marker of infectivity and the anti-HCV marker used to screen for HCV infection is a marker of exposure. Despite this, epidemiologically it will remain interesting to explore in future studies whether there are risk factors other than unsafe injection practices that are specifically affecting the spread of HCV infection in Pakistan.

## Conclusion

This study supports the general perception that HCV is growing in prevalence in the rural part of Pakistan. However, a lowering trend of HBV infection in literate blood donors is a welcome and interesting finding. There may be a need to have rural community based epidemiological studies to identify the determinants responsible for the spread of HCV infection and those that may be limiting the spread of HBV infection, particularly in the literate blood donor population, so that further preventative measures can be established.

## Competing interests

The author(s) declare that they have no competing interests.

## Authors' contributions

SAM made a substantial contribution to a conception, design, acquisition, analysis and interpretation of data. He remained involved in drafting the manuscript. MSP revised the analysis plan and draft critically and made an important intellectual contribution in the content.

## Pre-publication history

The pre-publication history for this paper can be accessed here:


